# Complete chloroplast genomes provide insights into evolution and phylogeny of *Zingiber* (Zingiberaceae)

**DOI:** 10.1186/s12864-023-09115-9

**Published:** 2023-01-18

**Authors:** Dongzhu Jiang, Xiaodong Cai, Min Gong, Maoqin Xia, Haitao Xing, Shanshan Dong, Shuming Tian, Jialin Li, Junyao Lin, Yiqing Liu, Hong-Lei Li

**Affiliations:** 1 College of Landscape Architecture and Life Science, Chongqing University of Arts and Sciences, Yongchuan, 402160 China; 2 College of Horticulture and Gardening, Yangtze University, Jingzhou, 433200 China; 3 College of Biology and Food Engineering, Chongqing Three Gorges University, Wanzhou, 404100 China; 4 Fairylake Botanical Garden, Shenzhen & Chinese Academy of Sciences, Shenzhen, 518004 China

**Keywords:** *Zingiber*, Chloroplast Genome, Phylogeny, Comparative genomics

## Abstract

**Background:**

The genus *Zingiber* of the Zingiberaceae is distributed in tropical, subtropical, and in Far East Asia. This genus contains about 100–150 species, with many species valued as important agricultural, medicinal and horticultural resources. However, genomic resources and suitable molecular markers for species identification are currently sparse.

**Results:**

We conducted comparative genomics and phylogenetic analyses on *Zingiber* species. The *Zingiber* chloroplast genome (size range 162,507–163,711 bp) possess typical quadripartite structures that consist of a large single copy (LSC, 86,986–88,200 bp), a small single copy (SSC, 15,498–15,891 bp) and a pair of inverted repeats (IRs, 29,765–29,934 bp). The genomes contain 113 unique genes, including 79 protein coding genes, 30 tRNA and 4 rRNA genes. The genome structures, gene contents, amino acid frequencies, codon usage patterns, RNA editing sites, simple sequence repeats and long repeats are conservative in the genomes of *Zingiber*. The analysis of sequence divergence indicates that the following genes undergo positive selection (*ccsA, ndhA, ndhB, petD, psbA, psbB, psbC, rbcL, rpl12, rpl20, rpl23, rpl33, rpoC2, rps7, rps12 and ycf3*). Eight highly variable regions are identified including seven intergenic regions (*petA-pabJ*, *rbcL-accD*, *rpl32-trnL-UAG*, *rps16-trnQ-UUG*, *trnC-GCA-psbM*, *psbC-trnS-UGA* and *ndhF-rpl32*) and one genic regions (*ycf1*). The phylogenetic analysis revealed that the sect. *Zingiber* was sister to sect. *Cryptanthium* rather than sect. *Pleuranthesis*.

**Conclusions:**

This study reports 14 complete chloroplast genomes of *Zingiber* species. Overall, this study provided a solid backbone phylogeny of *Zingiber*. The polymorphisms we have uncovered in the sequencing of the genome offer a rare possibility (for *Zingiber*) of the generation of DNA markers. These results provide a foundation for future studies that seek to understand the molecular evolutionary dynamics or individual population variation in the genus *Zingiber*.

**Supplementary Information:**

The online version contains supplementary material available at 10.1186/s12864-023-09115-9.

## Background

*Zingiber* Boehm. is a diverse genus of the family Zingiberaceae and consists of approximately 100–150 species that are widely distributed in the tropical and subtropical regions of Asia and Far East Asia [1, 2]. *Zingiber* contains many economically important species. Some species have long-lasting inflorescences and an assemblage of tightly clasped, brightly colored bracts and floral that often highly showy. They are widely used as landscaping and cut-flower in floral arrangements including chocolate pinecone ginger (*Z. montanum*) and Chiang Mai Princess (*Z. citriodorum*) [1–3]. In addition, some *Zingiber* species are widely cultivated as edible crop and among the best-known nonprescription drugs in traditional medicinal systems such as myoga ginger (*Z. mioga*), shampoo ginger (*Z. zerumbet*) and ginger (*Z. officinale*) [4–6]. Ginger have the pharmacological and biological potential effects of analgesic and anti-inflammatory, antibacterial, antitumor and antidiabetic [7–9]. In recent years, ginger was even considered as an alternative therapeutic agent for COVID-19 treatment based on its anti-viral activity [10–12].

The genus *Zingiber* could be distinguished based on nutritional and floral characteristics [1, 2]. Previous studies have shown that, species of *Zingber* can be divided into four groups, namely sect. *Zingiber*, sect. *Dymczewiczia*, sect. *Pleuranthesis* and sect. *Cryptanthium* based on the habit of inflorescences [13–15]. However, sect. *Dymczewiczia* was amalgamated with Sects. *Zingiber* and resolved as sister to sect. *Pleuranthesis* with weak support value according to the phylogenetic analysis of internal transcribed spacer (ITS) sequence of 23 *Zingiber* species and pollen morphology [16]. *Zingiber* species share similar characteristics of leaves and other vegetative organs, which makes it extremely difficult to identify species in the non-flowering stage [1–3]. Recently years, efforts have been made to explore the phylogenetic relationships among *Zingiber* species based on molecular data [16–19]. Kerss, et al. [17] found low resolution in identifying six *Zingiber* species using ITS and chloroplast *matK* regions. According to the analyses of amplified fragment length polymorphism (AFLP) DNA markers, *Z. montanum* was closely related to *Z. zerumbet* other than to *Z. officinale* [18]. These results were also revealed by Li, et al. [19] based on the complete chloroplast genome data. Overall, these previous studies have succeeded in clarifying the phylogenetic relationships of some *Zingiber* species, however, only small number of samples were used and the relationships among many species within the genus *Zingiber* are still unclear.

Chloroplast genomes have been used to address the chloroplast genome evolution, patterns and rates of nucleotide substitutions and phylogenetic relationships among land plants [20]. Chloroplast is a kind of vital organelle that can transform light energy into chemical energy in green plants [21, 22]. The chloroplast genome usually has a typical quadripartite structure consisting of a large single copy (LSC) region, a small single copy (SSC) region, and two copies of inverted repeats (IRs) shows and encodes 110–130 genes with a size range of 120–180 kb and [23–25]. In compare with mitochondrial and nuclear genome, chloroplast genome is typically inherited maternally and non-recombining [26]. Although the chloroplast genome structure is usually conserved in angiosperms, variations in genome size, genome structure, and gene substitution rate have been identified [27, 28]. In recent years, more than 40 complete chloroplast genomes have been sequenced in the family Zingiberaceae and divergent hotspots, which could be used for phylogenetic analyses, have been identified [25, 29–31]. However, only seven chloroplast genomes of *Zingiber* have been reported, which hindering the molecular plant identification and phylogenetic relationship clarification of *Zingiber* species. High throughput sequencing technology has made obtaining chloroplast genome sequences more practical and provides a unique opportunity to study the evolution of the chloroplast genome and the phylogeny of the genus *Zingiber*.

In this study, to characterize the genome structures, gene content, phylogeny and other characteristics of *Zingiber*, we sequenced chloroplast genomes of fourteen *Zingiber* species (Table 1). Then, we explored the molecular features of each genome and compared them with six other published chloroplast genomes within the *Zingiber*. Finally, we determined the chloroplast genome sequence variation, molecular evolution and phylogenetic relationships among 20 within the *Zingiber*.

**Table 1:** Summary features of complete chloroplast genomes of *Zingiber* species

Genome feature	*Zingiber cochleariforme*	*Zingiber densissimum*	*Zingiber* *ellipticum*	*Zingiber* *flavomaculosum*	*Zingiber koshunense*	*Zingiber* *leptorrhizum*	*Zingiber* *neotruncatum*
Genome size (bp)	163,665	163,607	163,455	163,298	163,394	162,956	162,484
LSC length (bp)	88,167	87,981	87,946	88,124	87,785	87,430	86,712
SSC length (bp)	15,788	15,846	15,771	15,644	15,835	15,722	15,812
IR length (bp)	29,855	29,890	29,869	29,765	29,887	29,902	29,957
GC content (%)							
Total genome	36.04%	36.08%	36.16%	36.12%	36.07%	36.18%	36.13%
LSC	33.78%	33.88%	33.92%	33.88%	33.84%	34.02%	33.95%
SSC	29.57%	29.40%	29.73%	29.68%	29.49%	29.61%	29.29%
IR	41.09%	41.07%	41.16%	41.14%	41.07%	41.07%	41.13%
Genes (total/different)	133/113	133/113	133/113	133/113	133/113	133/113	133/113
CDS (total/different)	87/79	87/79	87/79	87/79	87/79	87/79	87/79
tRNA (total/different)	38/30	38/30	38/30	38/30	38/30	38/30	38/30
rRNA (total/different)	8/4	8/4	8/4	8/4	8/4	8/4	8/4
Genes with introns	18	18	18	18	18	18	18
Different CDS in LSC	61	61	61	61	61	61	61
Different CDS in SSC	12	12	12	12	12	12	12
Different CDS in IRB	8	8	8	8	8	8	8
Different CDS in IRA	8	8	8	8	8	8	8
GenBank accession	OP869986	OP869975	OP869976	OP869987	OP869977	OP869984	OP869978

Genome feature	*Zingiber* *orbiculatum*	*Zingiber* *purpureum*	*Zingiber* *smilesianum*	*Zingiber* *striolatum*	*Zingiber* *xishuangbannaense*	*Zingiber* *yingjiangense*	*Zingiber* *Montanum*
Genome size (bp)	163,527	163,135	163,640	163,711	163,487	163,623	163,476
LSC length (bp)	88,032	87,730	88,142	88,026	88,199	87,957	87,797
SSC length (bp)	15,829	15,795	15,782	15,871	15,498	15,798	15,995
IR length (bp)	29,833	29,805	29,858	29,907	29,895	29,934	29,842
GC content (%)							
Total genome	36.06%	35.89%	36.07%	36.03%	36.04%	36.08%	35.90%
LSC	33.85%	33.66%	33.84%	33.82%	33.82%	33.88%	33.68%
SSC	29.39%	29.26%	29.53%	29.35%	29.71%	29.45%	29.27%
IR	41.10%	40.95%	41.09%	41.06%	40.95%	41.05%	40.93%
Genes (total/different)	133/113	133/113	133/113	133/113	133/113	133/113	133/113
CDS (total/different)	87/79	87/79	87/79	87/79	87/79	87/79	87/79
tRNA (total/different)	38/30	38/30	38/30	38/30	38/30	38/30	38/30
rRNA (total/different)	8/4	8/4	8/4	8/4	8/4	8/4	8/4
Genes with introns	18	18	18	18	18	18	18
Different CDS in LSC	61	61	61	61	61	61	61
Different CDS in SSC	12	12	12	12	12	12	12
Different CDS in IRB	8	8	8	8	8	8	8
Different CDS in IRA	8	8	8	8	8	8	8
GenBank accession	OP869979	OP869980	OP869981	ON646165	OP869982	OP869983	OP869985

*LSC* Lager single copy region, *SSC* Simple single copy region, *IR* Inverted repeat, *CDS* Protein coding gene

## Results

### Features of the *Zingiber* chloroplast genomes

**Fig. 1:**
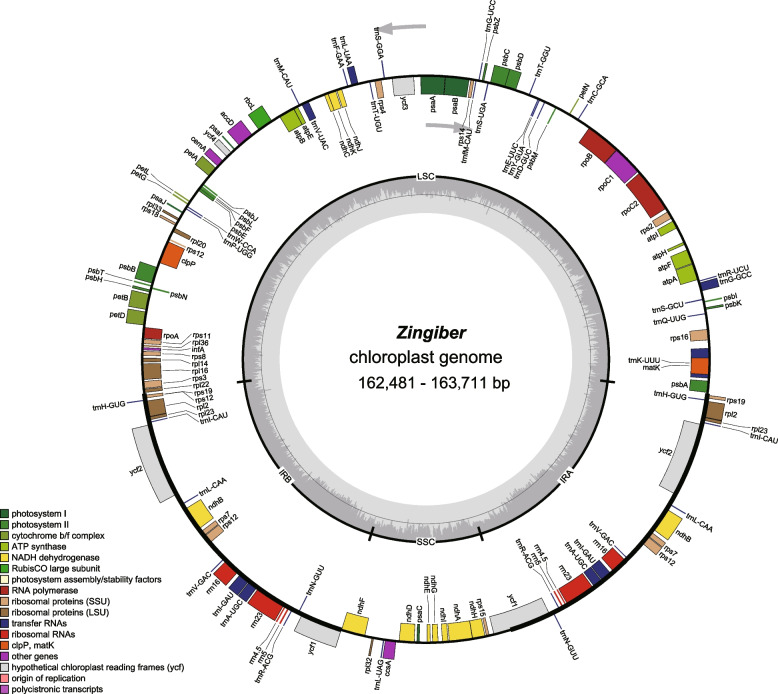
Chloroplast genome map of the genus *Zingiber* in this study. Genes belonging to different functional groups are shown in different colors in the outermost first ring. Genes shown on the outside of the outermost first ring are transcribed counter-clockwise and on the inside clockwise. The gray arrowheads indicate the direction of the genes. The tRNA genes are indicated by one letter code of amino acids with anticodons. LSC, large single copy region; IR, inverted repeat; SSC, small single copy region

All fourteen sequenced chloroplast genomes of *Zingiber* have a typical quadripartite structure containing one large single copy (LSC), one small single copy (SSC) and two inverted repeat regions (IRA and IRB) (Fig. 1, Table 1). The chloroplast genomes size of them ranged from 162,481 bp (*Z. neotruncatum*) to 163,711 bp (*Z. striolatum*), with an LSC region (86,988–88,199 bp) and an SSC region (15,498–15,995 bp) separated by two inverted repeat (IR) regions (29,765–29,934 bp). All fourteen chloroplast genomes show similar total GC content (35.89–36.18%), and the IR regions (40.93–41.16%) were significantly higher than the other two regions (Table 1, Fig. 1). The 14 sequenced chloroplast genomes contain 133 predicted functional genes, of which 113 were unique genes, including 79 protein coding genes, 30 tRNA genes, and 4 rRNA genes (Tables 1 and 2). Among the different protein coding genes in our fourteen sequenced chloroplast genomes, 61 genes are located in the LSC regions, 12 genes are located in the SSC regions, and 8 genes are duplicated in the IR regions (Table 1). There were 18 genes containing introns, most of them have only a single intron, whereas *ycf3* and *clpP* genes contain two introns (Table 2).

**Table 2:** Genes present in fourteen sequenced chloroplast genomes

Category for genes	Group of genes	Name of genes
Photosynthesis	Subunits of photosystem I	psaA, psaB, psaC, psaI, psaJ
	Subunits of photosystem II	psbA, psbB, psbC, psbD, psbE, psbF, psbH, psbI, psbJ, psbK, psbL, psbM, psbN, psbT, psbZ
	Subunits of cytochrome b/f complex	petA, petB^a^, petD^a^, petG, petL, petN,
	Subunits of ATP synthase	atpA, atpB, atpE, atpF^a^, atpH, atpI
	Subunits of NADH dehydrogenase	ndhA^a^, ndhB(×2)^a^, ndhC, ndhD, ndhE, ndhF, ndhG, ndhH, ndhI,
		ndhJ, ndhK
	Subunit of rubisco	rbcL
Self-replication	RNA polymerase	rpoA, rpoB, rpoC1^a^, rpoC2
	Large subunit of ribosomal proteins	rpl2(×2)^a^, rpl14, rpl16^a^, rpl20, rpl22, rpl23(× 2), rpl32, rpl33
		rpl36
	Small subunit of ribosomal proteins	rps2, rps3, rps4, rps7(×2), rps8, rps11, rps12(× 2)^a^, rps14,
		rps15, rps16^a^, rps18, rps19(×2)
	Ribosomal RNAs	rrn4.5(×2), rrn5(× 2), rrn16(× 2), rrn23(× 2)
	Transfer RNAs	trnA-UGC(×2)^a^, trnC-GCA, trnD-GUC, trnE-UUC, trnF-GAA, trnfM-CAU, trnG-GCC^a^, trnG-UCC, trnH-GUG(× 2), trnI-CAU (× 2), trnI-GAU(× 2)^a^, trnK-UUU^a^, trnL-CAA(× 2), trnL-UAA^a^, trnL-UAG, trnM-CAU, trnN-GUU(× 2), trnP-UGG, trnQ-UUG, trnR-ACG(× 2), trnR-UCU, trnS-GCU, trnS-GGA, trnS-UGA, trnT-GGU, trnT-UGU, trnV-GAC(× 2), trnV-UAC^a^, trnW-CCA, trnY-GUA
Other genes	Subunit of acetyl-coA-carboxylase	accD
	c-type cytochrome synthesis gene	ccsA
	Envelop membrane protein	cemA
	Protease	clpP^b^
	Translational initiation factor	infA
	Maturase	matK
Unknown function	Conserved open reading frames	ycf1(×2), ycf2(×2), ycf3^b^, ycf4

(×2): gene with two copies; ^a^gene containing one intron; ^b^gene containing two introns;

### Codon usage and RNA editing sites

**Fig. 2:**
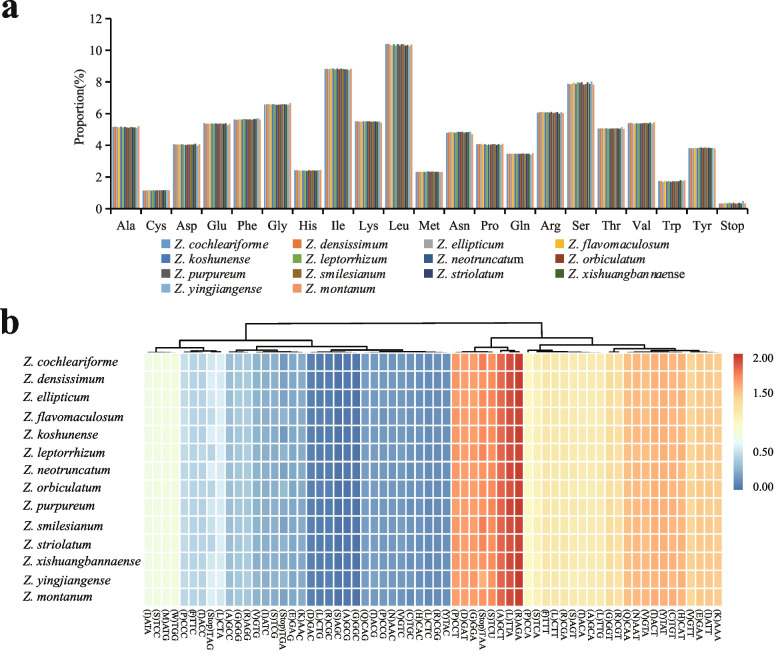
Codon content of all protein coding genes. **a** amino acids and stop codons proportion in protein coding sequences of fourteen sequenced chloroplast genomes and **b** heat map analysis for codon distribution of all protein coding genes of fourteen sequenced chloroplast genomes. Red colour indicates higher RSCU values and blue colour indicates lower RSCU values

Codon usage patterns and nucleotide composition help to lay a theoretical foundation for genetic modifications of the chloroplast genome [32]. A total of 79 protein coding genes in all 14 sequenced chloroplast genomes in *Zingiber* are analyzed for codon usage frequency. They comprise 25,557 (*Z. montanum*) to 26,354 (*Z. xishuangbannaense*) codons. Of the 25,557–26,354 codons, leucine (Leu) is the most abundant amino acid, with a frequency of 10.25–10.40%, followed by isoleucine (Ile) with a frequency of 8.75–8.85%, while cysteine (Cys) is the least common, with a frequency of 1.14–1.18% (Fig. 2a). Because of the value of relative synonymous codon usage (RSCU) > 1.00, thirty codons show codon usage bias in protein coding genes of the 14 sequenced chloroplast genomes. Stop codon usage is biased toward TAA (RSCU > 1.00) (Fig. 2b). Both methionine (Met) and tryptophan (Trp) exhibit no codon bias and have RSCU values of 1.00 (Fig. 2b).

Furthermore, 72–81 RNA editing sites were identified in 27 protein-coding genes of 14 chloroplast genomes, with the least in *Z. montanum* (72 sites) and *Z. purpureum* (72 sites), and the most in *Z. orbiculatum* (81 sites) (Table S1). In the 14 identified chloroplast genomes that we sequenced, the *ndhB* gene has the highest number of potential editing sites (11 sites), followed by the *ndhD* gene (7 sites) (Table S1). All of these editing sites are C-to-T transitions that occur at the first or second positions of the codons.

### Features of simple sequence repeats (SSRs) and long repeats

**Fig. 3:**
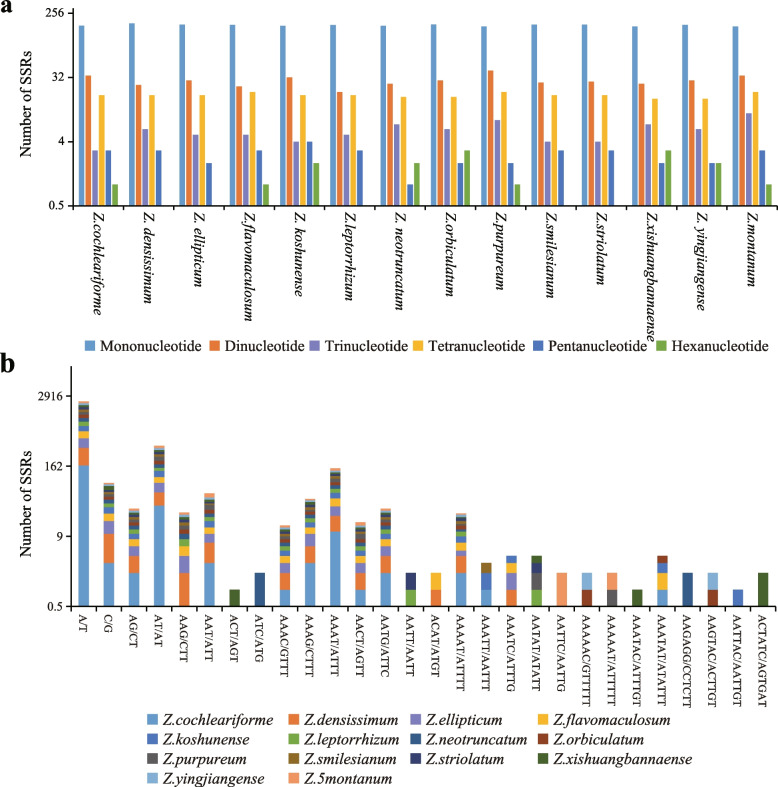
Comparison of the simple sequence repeats (SSRs) among fourteen *Zingiber* species. **a** the number of different SSR types. **b** the frequency of the identified SSRs in different repeat class types

A total number of 221 to 238 SSRs were identified in all sequenced chloroplast genome. (Fig. 3). Among each sequenced chloroplast genome, mononucleotide repeats were the most frequent, with numbers ranging from 167 to 184, which accounted for 70.18–79.09% of all SSRs, followed by dinucleotide, ranging from 24 to 40 (9.09–16.81%), tetranucleotide, ranging from 16 to 20 (6.96–8.77%), trinucleotide, ranging from 3 to 10 (1.30–4.26%), pentanucleotide, ranging from 1 to 4 (0.45–1.74%), and hexanucleotide, ranging from 0 to 3 (0–1.36%). The majority of the mononucleotide SSRs were A/T repeats, which accounted for 68.07–75.00% of all the repeat types among the fourteen sequenced chloroplast genomes, followed by AT/AT repeats, ranging from 8.18–15.97%, and the remaining repeat types below 6% (Fig. 3b).

**Fig. 4:**
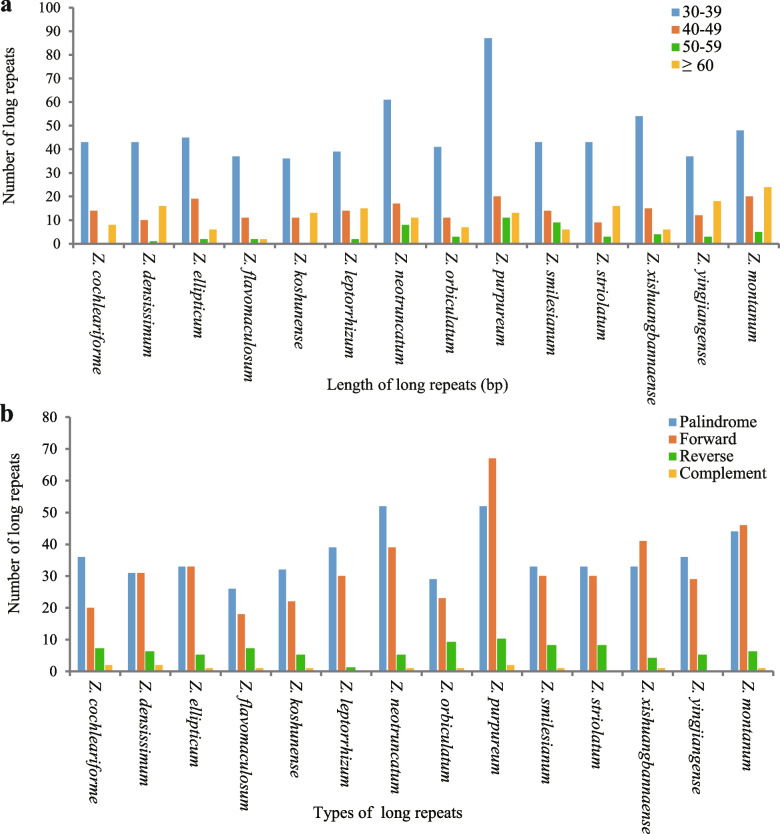
Long repeat sequences among fourteen *Zingiber* species. **a** total of four long repeat types in fourteen chloroplast genomes and **b** numbers of long repeat sequences by length

Long repeats that longer than 30 bp may have the function of promoting chloroplast genome rearrangement and increasing population genetic diversity, which has been a hotspot in genomic research [33]. In this study, 14 sequenced chloroplast genomes had 1068 long repeats that consisted of 509 palindromic repeats, 459 forward repeats, 86 reverse repeats, 14 complement repeats, 86 reverse repeats (Fig. 4a). *Z. montanum* had the largest number (131), and Z. *flavomaculosum* had the smallest number of long repeats (52) (Fig. 4a). In addition, the numbers of the four repeat types are quite different in *Zingiber*, with palindromic repeats and forward repeats a clear quantitative superiority (20–67), while complement repeats and reverse repeats are less abundant (0–10) (Fig. 4b). Moreover, among all long repeats, most sequences were between 30 and 39 bp (657) in length, followed by 40–49 bp (197), and 50–59 bp had the least number (53) (Fig. 4b).

### Contraction and expansion of inverted repeats (IRs)

**Fig. 5:**
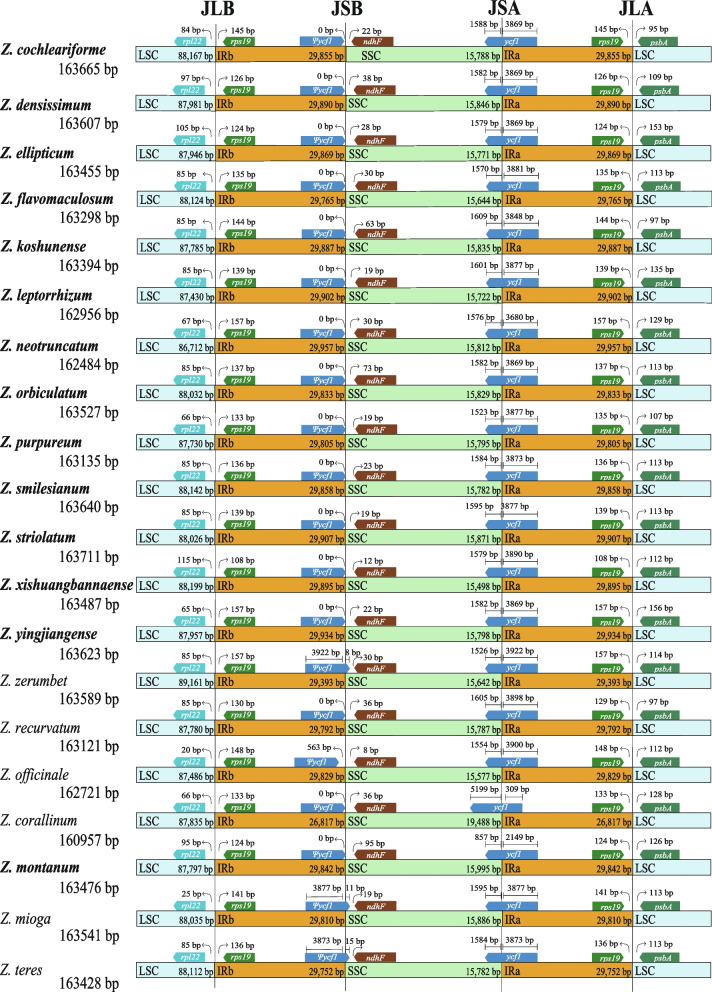
Comparisons of LSC, SSC and IR regions boundaries among 20 chloroplast genomes. Ψ: pseudogenes

A comprehensive comparison at the LSC/IRs/SSC boundaries was performed among the 20 *Zingiber* species (Fig. 5). Although the inverted repeat regions (IRA and IRB) are the most conserved regions of the chloroplast genome, shrinkage and expansion of the IR boundaries are hypothesized to help explain size differences between chloroplast genomes beyond genus. The length of the IR region in the 14 chloroplast genomes exhibited a modest expansion, ranging from 29,765 bp to 29,957 bp. Within the 20 chloroplast genomes of *Zingiber* species, the *rpl22* and *rps19* genes were located in the boundaries of the LSC/ IRB regions (Fig. 5). There were 20–115 bp between *rpl22* and the LSC/IRB borders, and the distance between *rps19* and the LSC/IRB boundary ranged from 108 bp to 157 bp (Fig. 5).

*Ψycf1-ndhF* genes were located at the boundaries of the IRB/SSC regions in all 20 *Zingiber* species. The IRB/SSC borders of 8 species (*Z. cochleariforme*, *Z. densissimum*, *Z. ellipticum*, *Z. flavomaculosum*, *Z. orbiculatum*, *Z. yingjiangense*, *Z. recurvatum* and *Z. corallinum*) were all situated adjacent to the end of *Ψycf1*. In addition, *Ψycf1* expanded into the SSC regions in 8 species, namely, *Z. koshunense*, *Z. purpureum*, *Z. smilesianum*, *Z. xishuangbannaense*, *Z. zerumbet*, *Z. montanum*, *Z. mioga* and *Z. teres*, for 25 bp, 5 bp, 9 bp, 1 bp, 8 bp, 83 bp, 11 bp and 15 bp, respectively (Fig. 5). There were 63 bp, 19 bp, 23 bp, 12 bp, 30 bp, 95 bp, 19 bp and 15 bp between the *ndhF* and LSC/IRB borders in *Z. koshunense*, *Z. purpureum*, *Z. smilesianum*, *Z. xishuangbannaense*, *Z. zerumbet*, *Z. montanum*, *Z. mioga* and *Z. teres*, respectively (Fig. 5).

The SSC/IRA boundary was situated in the *ycf1* coding region, which crossed into the IRA region in all 20 *Zingiber* species. However, the length of *ycf1* in the IRA region varied among the 20 *Zingiber* species from 309 bp to 3922 bp (Fig. 5).

The *rps19* and *psbA* genes were situated in the boundaries of the IRA/LSC regions in all 20 *Zingiber* species, in which the distances between *rps19* and the IRA/LSC border ranged from 108 bp to 157 bp (Fig. 5). For all 20 *Zingiber* species, a 95–156 bp distance was observed between the *psbA* gene and the IRA/LSC border (Fig. 5).

### Genomic comparative and nucleotide diversity analyses

**Fig. 6:**
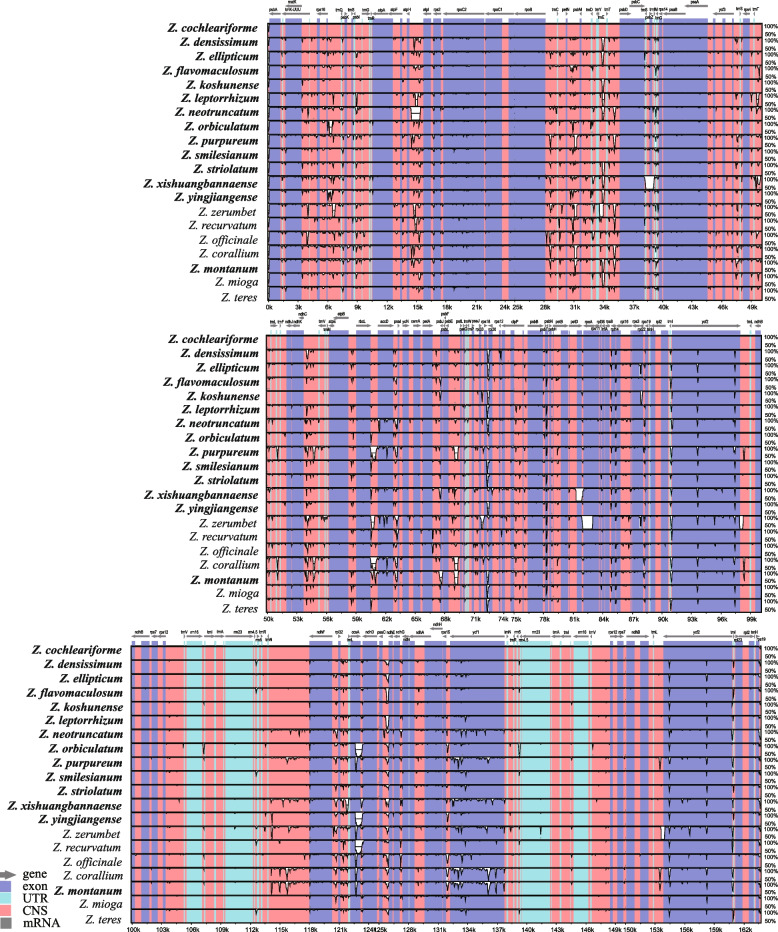
Comparative plots of percent sequence identity of 20 chloroplast genomes in *Zingiber.* Coarse species represent chloroplast genome obtained in this study

Multiple alignments of 20 *Zingiber* chloroplast genomes were compared by mVISTA, with the annotated *Z. cochleariforme* genome sequence as the reference (Fig. 6). The mVISTA comparison showed that the LSC and SSC regions were more divergent than the two IR regions. Moreover, the non-coding region exhibited more nucleotide divergence than the coding regions. The main divergences for the coding regions were located in the region of *accD*, *ccsA, rpoC2* and *ycf1*. For the non-coding regions, strongly divergent regions were *rbcL-accD*, *trnT-UGU-trnL-UAA*, *rps16-trnQ-UUG*, *atpI-atpH*, *petN-psbM*, *trnT-UGU-trnL-UAA*, *ndhF-rpl32*, *rpl32-trnL-UAG*, *trnN-ndhF* and *trnL-ycf1* (Fig. 6).

**Fig. 7:**
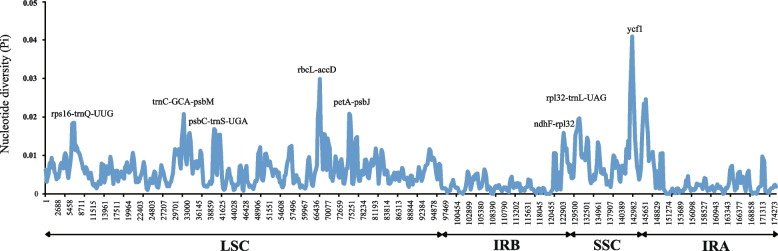
Nucleotide diversity (Pi) values of various regions in 20 chloroplast genomes

Furthermore, nucleotide diversity (Pi) values were calculated within 800 bp windows (Fig. 7) to identify sequence divergence hotspots. The results showed that the Pi value of the whole *Zingiber* chloroplast genome varied from 0 to 0.04088. Eight highly variable regions (Pi> 0.016) were detected: *petA-pabJ*, *rbcL-accD*, *rpl32-trnL-UAG*, *rps16-trnQ-UUG*, *trnC-GCA-psbM*, *psbC-trnS-UGA* and *ndhF-rpl32* and *ycf1*. Among these, five regions (*petA-pabJ*, *rbcL-accD*, *rps16-trnQ-UUG*, *trnC-GCA-psbM* and *psbC-trnS-UGA* were located in the LSC region, and the remaining three were in the SSC region (Fig. 7). This is consistent with preceding results that the IR region is generally more conserved than the LSC and the SSC regions.

### Characterization of substitution rates and positive selection analyses

The non-synonymous (dN) and synonymous (dS) substitution rates of all 79 protein coding genes were analyzed across 20 *Zingiber* species. Most of the genes were subjected to purifying selection. Using the likelihood ratio test, we found that 19 protein coding genes were under positive selection with posterior probability greater than 0.95 (Table 3). Among the 19 protein coding genes, *ycf1* showed the highest number of positive amino acids sites (52), followed by *ycf2* (24) and *clpP* (12) (Table 3). The other 16 protein coding genes, *ccsA*, *ndhA*, *ndhB*, *petD*, *psbA*, *psbB*, *psbC*, *rbcL*, *rpl12*, *rpl20*, *rpl23*, *rpl33*, *rpoC2*, *rps7*, *rps12* and *ycf3*, presented 2, 5, 3, 1, 2, 1, 2, 11, 1, 5, 1, 5, 3, 1, 1 and 1 amino acids sites were truly under positive selection respectively (Table 3).

**Table 3:** Positive selective amino acid loci and estimation of parameters

Gene	Ln L	Estimates of parameters	Positively selected sites
ccsA	− 1688.900532	p0 = 0.99053p = 0.00799q = 0.00500 (p1 = 0.00947)ω = 122.48812	200 F 0.999**, 201 I 0.986*
clpP	− 1189.046814	p0 = 0.91790p = 0.02800q = 0.01097 (p1 = 0.08210)ω = 22.05525	22 E 0.966*, 24 Y 1.000**, 39 K 0.972*, 40 E 0.969*, 58 Q 0.979*, 72 W 0.979*, 75 S 0.998**, 78 A 0.960*, 120 V 1.000**, 153 T 0.975*, 192 L 0.961*, 204 L 0.953*
ndhA	− 1741.334638	p0 = 0.98308p = 12.72091q = 99.00000 (p1 = 0.01692)ω = 52.52860	132 S 0.996**, 189 R 1.000**, 190 V 1.000**, 191 I 0.998**, 192 L 0.998**
ndhB	− 2096.337425	p0 = 0.98968p = 0.00500q = 1.58164 (p1 = 0.01032)ω = 999.00000	133 I 0.995**, 145 G 0.951*, 181 T 0.995**
petD	− 753.029169	p0 = 0.96547p = 0.00500q = 1.79217 (p1 = 0.03453)ω = 7.43965	8 I 0.970*
psaA	− 3396.235357	p0 = 0.99301p = 0.00500q = 0.75911 (p1 = 0.00699)ω = 41.45919	261 F 0.999**, 292 A 0.981*
psbB	− 2254.262995	p0 = 0.99605p = 0.00500q = 1.52674 (p1 = 0.00395)ω = 373.49395	488 A 1.000**
psbC	− 2130.498976	p0 = 0.99356p = 0.00500q = 1.59626 (p1 = 0.00644)ω = 85.67788	24 T 0.970*, 280 S 0.978*
rbcL	− 2275.465641	p0 = 0.96928p = 0.48224q = 5.55236 (p1 = 0.03072)ω = 24.32347	19 G 0.992**, 30 T 0.999**, 176 L 0.988*, 232 L 1.000**, 233 F 1.000**, 254 C 0.981*, 269 T 0.970*, 289 H 0.998**, 333 I 0.998**, 431 L 0.987*, 456 S 1.000**
rpl12	− 1180.117502	p0 = 0.99203p = 0.00500q = 3.98754 (p1 = 0.00797)ω = 77.23012	140 S 0.966*
rpl20	− 745.824555	p0 = 0.95179p = 0.00871q = 0.02374 (p1 = 0.04821)ω = 35.61813	120 S 1.000**, 121 N 1.000**, 122 K 1.000**, 123 V 1.000**, 124 H 1.000**
rpl23	−430.542995	p0 = 0.97858p = 0.00500q = 2.07543 (p1 = 0.02142)ω = 999.00000	57 E 0.976*
rpl33	− 288.922817	p0 = 0.00001p = 0.00500q = 2.19296 (p1 = 0.99999)ω = 999.00000	3 K 0.988*, 6 D 0.988*, 29 G 0.988*, 42 M 0.988*, 43 P 0.988*
rpoC2	− 6439.688674	p0 = 0.99492p = 0.04204q = 0.16546 (p1 = 0.00508)ω = 22.05755	593 D 0.978*, 743 P 0.985*, 1181 W 0.986*
rps7	− 698.656393	p0 = 0.98065p = 0.00500q = 1.44246 (p1 = 0.01935)ω = 485.63912	81 G 0.966*
rps12	−575.913647	p0 = 0.99188p = 0.01544q = 0.03483 (p1 = 0.00812)ω = 999.00000	116 Q 0.958*
ycf1	−10,914.54603	p0 = 0.70387p = 0.00500q = 1.91338 (p1 = 0.29613)ω = 7.28992	14 S 0.996**, 16 I 0.990*, 48 R 0.970*, 65 I 0.994**, 315 R 0.958*, 353 R 0.977*, 384 S 0.961*, 404 L 0.994**, 464 E 0.952*, 588 N 0.997**, 598 F 0.994**, 622 E 0.990*, 682 L 0.953*, 684 A 0.994**, 699 H 0.994**, 705 Q 0.996**, 728 S 1.000**, 729 V 0.997**, 748 Q 0.996**, 753 R 1.000**, 861 I 0.998**, 869 L 0.961*, 883 L 0.965*, 900 Y 0.972*, 905 E 0.951*, 936 T 0.991**, 944 L 0.952*, 982 T 0.960*, 991 A 0.968*, 1027 P 0.991**, 1339 R 0.996**, 1449 R 0.997**, 1538 I 0.995**, 1539 S 0.996**, 1547 H 0.990**, 1564 S 1.000**, 1568 W 0.979*, 1569 S 0.977*, 1597 F 0.962*, 1657 T 0.991**, 1671 P 0.965*, 1672 L 0.956*, 1690 S 0.996**, 1695 L 0.953*, 1713 I 0.994**, 1715 H 0.993**, 1719 R 0.990*, 1748 L 0.996**, 1750 A 1.000**, 1758 T 0.954*, 1759 L 0.997**, 1780 G 0.999**
ycf2	−10,302.30873	p0 = 0.96727p = 5.07671q = 3.06985 (p1 = 0.03273)ω = 65.06865	847 R 0.994**, 1008 D 1.000**, 1087 G 0.993**, 1353 L 1.000**, 1390 T 0.993**, 1417 E 0.993**, 1421 S 0.994**, 1461 P 0.994**, 1654 H 0.993**, 1677 I 0.993**, 2117 R 0.994**, 1997 T 0.993**, 2117 R 0.994**, 2231 Q 0.994**, 2247 R 1.000**, 2317 L 0.972*, 2319 H 0.993**, 2321 T 0.999**, 2322 G 0.995**, 2323 E 0.993**, 2324 R 0.993**, 2325 F 0.999**, 2327 I 0.993**, 2328 P 0.994**
ycf3	− 735.294401	p0 = 0.98001p = 0.00500q = 1.68882 (p1 = 0.01999)ω = 32.38804	44 M 0.998**

The degree of freedom for each gene was 38; * and ** indicate posterior probability higher than 0.95 and 0.99, respectively

### Phylogenetic analyses

**Fig. 8:**
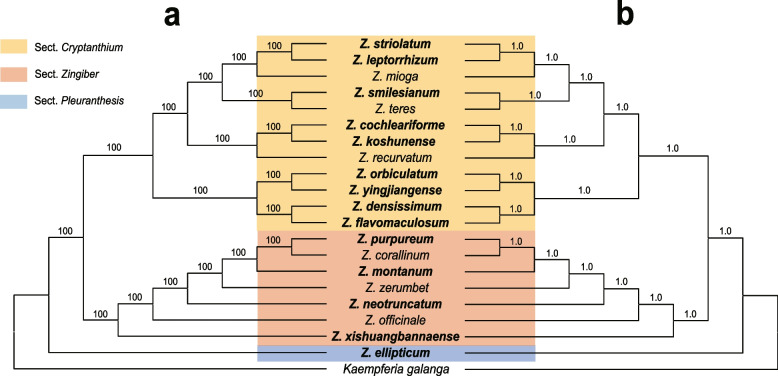
Molecular phylogenetic tree based on 20 chloroplast genomes within the genus *Zingiber*. **a** Maximum likelihood tree. **b** Bayesian tree. Coarse species represent chloroplast genome obtained in this study

The phylogeny of 55 Zingiberaceae species were well resolved (Fig. S1). *Zingiber* is monophyletic (BS = 100%) and was well resolved as sister to *Kaempferia* with strong support (BS = 100%). Based on the chloroplast genome dataset, we generated a well-resolved phylogeny of *Zingiber* (Fig. 8). The support values of all the branches in both ML and BI trees were robust (BI = 1.0, BS = 100%). Thus, we will not include the support values in the text below. *Zingiber* was divided into three sections: sect. *Crytanthium*, sect. *Zingiber*, and sect. *Pleuranthesis*. Sect. *Crytanthium* is resolved as sister to sect. *Zingiber*. There are four major clades of the sect. *Crytanthium*. The first branch was well supported and comprised *Z. flavomaculosum* + *Z. densissimum* as sister to *Z. yingjiangense* + *Z. orbiculatum*. The second clade was *Z. recurvatum* + (*Z. koshunense* + *Z. cochleariforme*)*.* Within the rest of the sect. *Cryanthium*, two subclades were recovered: *Z. teres* + *Z. smilesianum* and *Z. mioga* + (*Z. leptorrhizum* + *Z. striolatum*). In sect. *Zingiber*, *Z. xishuangbannaense*, subsequently followed by *Z. officinale*, *Z. neotruncatum*, *Z. zerumbet*, *Z. montanum* was sister to *Z. corallinum* + *Z. purpureum*. As for sect. *Pleuranthesis*, which contains only one species (*Z. ellipticum*).

## Discussion

In this study, 14 *Zingiber* chloroplast genomes were newly reported. Their genome size (162,481 bp-163,711 bp), GC content (35.89–36.18%), genome quadripartite structure, gene composition, all of the protein-coding genes, tRNA and rRNA showed high similarity, which were in consistent with other Zingiberoideae chloroplast genomes [25, 29–31]. The conservation of plastomes had been observed in various angiosperms such as Malvaceae, Araceae in which the same gene content and gene order had been reported [34–37]. Nevertheless, plastome rearrangement, gene duplication, gene loss and intron loss are reported in a number of plant lineages [22, 25, 38]. Although structure variations occurred in some Zingiberoideae plants for example, both *trnS-GGA* and *trnT-GGU* were lost in the chloroplast genome of *Globba schomburgkii*. The *lhbA* gene were lost in both *Hedychium coccineum* and *Hedychium neocarneum* [25]. However, the chloroplast genomes of *Zingiber* species were highly conserved in current study, which is in agreement with previous studies at genus level *Camellia* [39], *Sinosenecio* [40], and *Chrysosplenium* [41]. Plastomes are very conservative which was maintained by multiple molecular mechanisms including uniparental inheritance, rarity of plastid fusion, and the presence of an active repair mechanism [21, 35]. Hence, the typically conservative nature of the *Zingiber* plastomes is linked to a certain molecular mechanism.

The expansion and contraction at the borders of the IR regions of chloroplast genomes is common in angiosperms, which may cause size variations, gene duplication or the reduction, and the origination of pseudogenes [20, 42, 43]. Abnormal expansion of IR regions had been observed in some taxon e.g., *Pilea* [44], *Erodium* [45] and *Pelargonium* [46], which transferred numerous genes from the SC regions into the IR. In this study, we found that expansion and contraction of IRs showed much similarity among the species of the genus *Zingiber*, and the distribution and locations of gene types in these regions were highly consistent. These results are in agreement with previous report of Zingiberoideae [25]. The IR/SSC boundary shifts always cause the increased length in the IR regions. Here, we found the IR/SC boundary of *Zingiber* is relatively stable. The pseudogene of *ycf*1 originated at the junction of IR in *Zingiber* plants which was also observed in other angiosperms [25, 35]. Compared with the chloroplast genomes of six *Zingiber* species published in NCBI, the length of IR region of all species assembled by ourselves was basically the same, and no gene loss was detected. Overall, the conservation of the IR of the *Zingiber* plants may be one of the reasons for its stability in length and structure.

Highly variable regions are always used as DNA barcode markers for the studies on species identification and phylogenetic analyses. The high similarity of the vegetative characteristics has made it extremely difficult to distinguish *Zingiber* plants [16]. Since some classical DNA barcodes are insufficient for species identification and phylogeny of *Zingiber*, it is very important to find more highly variable regions at genus level that could be developed as representing potential markers for future variety identification research. Based on the results of mVISTA and nucleotide diversity, eight highly variable regions among 20 *Zingiber* species are identified including seven intergenic regions (*petA-pabJ*, *rbcL-accD*, *rpl32-trnL-UAG*, *rps16-trnQ-UUG*, *trnC-GCA-psbM*, *psbC-trnS-UGA* and *ndhF-rpl32*) and one genic region (*ycf1*). These highly variable regions could be used as potential DNA barcode for species identification and phylogenetic analysis for the *Zingiber* species. Among them, *ndhF-rpl32*, *rpl32-trnL-UAG* and *ycf1* were reported as suitable for species identification at subfamily and genus level in Zingiberoideae [25]. The *ycf1* gene is the most variable site in the chloroplast genome, showing greater variability than existing chloroplast candidate barcodes such as *matK* and *rbcL* [47]. Other five intergenic regions that identified in the present study are also reported in other plants at species level. For example, *petA-pabJ* was demonstrated well utilization as DNA barcodes for *Lindera* plant [48] and *rbcL-accD* was identified to be an effective marker for *Rumex* species [49]. Sun, et al. [50] suggested that *petA-psbJ*, *ndhF-rpl32* and *rpl32-trnL* potentially be used as molecular genetic markers for population genetics and phylogenetic studies of *Magnolia polytepala*. And *rps16-trnQ-UUG*, *trnC-GCA-psbM*, *psbC-trnS-UGA* are also reported in previous studies [51, 52]. Generally, although several candidate barcoding regions were identified, further research is still necessary to determine whether these highly divergent markers could be used in the identification and phylogenetic analyses of *Zingiber* species.

Positive selection is assumed to play key roles in the adaptation of organisms to diverse environments [53], while negative (purifying) selection is a ubiquitous evolutionary force responsible for genomic sequence conservation across long evolutionary timescales [54]. In this study, 19 genes with positive selection sites are identified in *Zingiber*. Among these genes containing amino acid positive sites, we found that *ycf1* and *ycf2* genes possess higher number (52, 24, respectively) of positive amino acid sites within *Zingiber* species, suggesting that the *ycf1* gene may play important roles in the adaptive evolution of *Zingiber* species. Six genes (*rpl12*, *rpl20*, *rpl23*, *rpl33*, *rps7* and *rps12*) encoding ribosomal subunit proteins are under positive selection, and these genes are considered to be essential for chloroplast biogenesis and function, suggesting that *Zingiber* plants may increase the adaptability of evolution by regulating encoding ribosomal subunit proteins in chloroplasts [55]. Moreover, eleven genes, namely *ccsA*, *clpP*, *ndhA*, *ndhB*, *petD*, *psaA*, *psbB*, *psbC*, *rbcL*, *rpoC2* and *ycf3*, have also been identified with positive selection sites in current study. Recent studies have indicated that these nineteen genes with positive selection in some angiosperms are common. For examples, *ccsA*, *rbcL*, *rpoC2* have been identified under positive selection in Orchidaceae, *Euterpe*, and *Pterocarpus* [24, 56, 57]; In Zingiberoideae, *ccsA*, *ndhA*, *ndhB*, *psbJ*, *rbcL*, *rpl20*, *rpoC1*, *rpoC2*, *rps12*, *rps18*, *ycf1*, *ycf2* and *ycf4* have also been identified under positive selection [25]. *Zingiber* species mainly inhabited warm, humid, semi-shaded environment and maintain a high level of plant diversity [1, 3]. Therefore, based on our analyses, we believe that positive selection of these chloroplast genes may be promote the adaptation of *Zingiber* plants to semi-shaded environment, but the detailed adaptation mechanism needs further in-depth research.

The phylogenetic analysis of 55 Zingiberaceae species showed that *Zingiber* was well resolved as sister to *Kaempferia* with strong support (Fig. S1), which is consistent with previous studies [17, 19, 25, 58–60]. Previously, the classification of *Zingiber* species was usually based on the type of inflorescence and pollen morphology, which generally solved the classification problems of *Zingiber* plants [61]. *Zingiber* was classified into three sections based on ITS sequences analyses together with similarity in pollen morphology and inflorescence habit [16, 17]. Our species-level phylogenetic tree of *Zingiber* showed that three traditionally accepted sections were monophyletic with strong support. In different with the result of Theerakulpisut [16] based on the ITS analyses, our results strongly supported sect. *Cryptanthium* as sister to sect *Zingiber* rather than sect. *Pleuranthesis*. Conflicts between phylogenetic trees delineated by chloroplast genomes and nuclear genes are also common in some angiosperms, such as Asteraceae and Zingiberaceae [62–68]. The conflict phenomenon may be due to reticulate evolution in the events of rapid diversification or uniparental inheritance of the plastome [35, 62]. However, the mechanism that leads to the conflict in *Zingiber* require further in-deep research. Additionally, the phylogeny indicated strong support for interspecies relationships. In sect. *Zingiber*, *Z. purpureum* was well resolved as sister to *Z. corallinum*. *Z. xishuangbannaense*, a species endemic to china, was resolved as the first lineage split from *Zingiber* in this study. The reminder *Zingiber* species formed a monophyletic clade with strong support, which is consistent with previous studies [16, 25]. The rest of the sect. *Zingiber* formed a strong supported clade. Although Theerakulpisut, et al. [16] recognized this clade, but the bootstrap value is below 50% and relationships among a number of lineages of this clade are uncertain. Our results demonstrated that *Z. neotruncatum* subsequently followed by *Z. zerumbet* was sister to *Z. montanum* + (*Z. corallinum* + *Z. purpureum*). For sect. *Cryptanthium*, 12 species, including 9 newly sequenced species in this study, were sampled, which is the mostly densest sampling to date. The relationships among lineages of sect. *Cryptanthium* were well resolved with robust support and provided a back bone for further classification at the infrageneric level and for investigating the biogeography of this group.

## Conclusions

In this study, fourteen complete chloroplast genomes of *Zingiber* species have been sequenced, assembled and annotated for the first time. The structural characteristics of these fourteen chloroplast genomes are shown to be conservative, which are similar to those reported chloroplast genomes of Zingiberoideae species. Meanwhile, comparative analyses of 20 *Zingiber* chloroplast genomes have generated 8 highly variable regions, which may be used as a potential source of molecular markers for species identification. Based on whole chloroplast genomes data, phylogenetic relationships among 20 *Zingiber* species have been clearly resolved. We found sect. *Cryptanthium* as sister to sect *Zingiber* rather than to sect. *Pleuranthesis*. The conflict phenomenon may be due to reticulate evolution in the events of rapid diversification or uniparental inheritance of the plastome. In addition, 19 genes are under positive selection with high posterior probabilities, which may play important roles in *Zingiber* species adaption to semi-shaded environment. Overall, our research has greatly enriched the genome resources of *Zingiber*, which will help to further analyze the phylogeny of *Zingiber* and resolve the genetic relationships within *Zingiber* in the future.

## Materials and methods

### Plant material, DNA extraction, and sequencing

A total of 21 chloroplast genomes were used for this study, including seven chloroplast genomes obtained from GenBank (www.ncbi.nlm.nih.gov/genbank) and fourteen newly generated in this study (Table 1). Genomic DNA was isolated from silica-gel dried leaf tissue or herbarium specimens (Table S2) using Plant Genomic DNA Kit (TIANGEN, Beijing, China), The concentration and quantity of each isolated genomic DNA sample were determined with a NanoDrop 2000 micro spectrometer (Wilmington, DE, USA) and 1% agarose gel electrophoresis, respectively. DNA was used to construct PE libraries with insert sizes of 150 bp and sequenced by the MGI DNBSEQ-T7 platform (MGI-TECH, Shen Zhen, China).

### Chloroplast genome assembly and annotation

For each accession, 5.0 Gb raw data were generated with pair-end 150 bp read length. Trimmomatic v0.39 [69] was used to remove low-quality and adapter-containing reads. The clean data were then assembled using GetOrganelle v1.7.5 [70]. The assembled chloroplast genomes were annotated in Geneious R11 with *Z. officinal* (MW602894), *Z. teres* (NC_062457), *Z. mioga* (NC_057615), *Z. recurvatum* (MT473712) and *Z. zerumbet* (MK262726) as references, and then manually checked for start/stop codons. Finally, the OGDRAW v1.3.1 program was used to draw the circular chloroplast genome maps of the *Zingiber* species with default settings.

### Codon usage and RNA editing sites

Codon usage patterns and nucleotide composition could help to lay a theoretical foundation for genetic modifications of the chloroplast genome [32]. Here, to examine the deviation in synonymous codon usage, the relative synonymous codon usage (RSCU) was calculated using the software CodonW (University of Texas, Houston, TX, USA) with the RSCU value (Fig. 2a). When the RSCU value > 1.00, it means that the use of a codon is more frequent than expected, and vice versa. The clustered heat map of RSCU values of fourteen sequenced *Zingibe*r chloroplast genomes was conducted by R v3.6.3 (https://www.R-project.org.) (Fig. 2b). To predict possible RNA editing sites in the twenty chloroplast genomes, protein coding genes were used to predict potential RNA editing sites using the online program Predictive RNA Editor for Plants (PREP) suite (http://prep.unl.edu/) with a cut of value of 0.8.

### Analyses of SSRs and long repeats

Chloroplast SSR has high variation level within the same species and is an important source for developing molecular markers, which are widely used in phylogenetic and population genetic analysis [71]. MIcroSAtellite (MISA) (http://pgrc.ipk-gatersleben.de/misa/) was used to detect the simple sequence repeat (SSRs or microsatellites) motifs in fourteen sequenced chloroplast genomes with the settings as follows: 8 for mono-, 5 for di-, 4 for tri-, and 3 for tetra-, pena-, and hexa-nucleotide SSRs (Fig. 3). The REPuter software was employed to identify long repeats such as forward, palindrome, reverse and complement repeats. The criteria for determining long repeats were as follows: (1) a minimal repeat size of more than 30 bp; (2) a repeat identity of more than 90%; and (3) a hamming distance equal to 3 (Fig. 4).

### Genome comparison and nucleotide variation analysis

To detect the contractions and expansions of the IR regions in the chloroplast genomes of the *Zingiber*, 20 whole genomes within *Zingiber* were compared (Fig. 5). The online software mVISTA tool with the Shufe-LAGAN mode [72] was used to make pairwise alignments among these 20 whole chloroplast genomes with the annotated chloroplast genome of *Z. cochleariforme* as reference (Fig. 6). The 20 chloroplast genomes of *Zingiber* were first aligned using MAFFT v7 [73] and then manually adjusted using BioEdit v7.0.9 [74]. DnaSP v5.10 software [75] was used to calculate the nucleotide variability (Pi) of the 20 chloroplast genomes within the *Zingiber*, with a sliding window analysis with the step size and window length set as 200 bp and 800 bp (Fig. 7).

### Positive selection analysis

To identify the genes under selection, we scanned the chloroplast genomes of fourteen species within *Zingiber* using the software EasyCondeML [76]. The software was used for calculating the non-synonymous (dN) and synonymous (dS) substitution rates, along with their ratios (ω = dN/dS). The analyses of selective pressures were conducted along the ML tree of these fourteen species in Newick format. Each single-copy CDS sequences was aligned according to their amino acid sequence. The site-specific model with five site models (M0, M1a & M2a, M7 & M8) were employed to identify the signatures of adaptation across chloroplast genomes. This model allowed the ω ratio to vary among sites, with a fixed ω ratio in all the branches. The site-specific model, M1a (nearly neutral) vs. M2a (positive selection) and M7 (β) vs. M8 (β & ω) were calculated in order to detect positive selection [77]. Likelihood ratio test (LRT) of the comparison (M1a vs. M2a and M7 vs. M8) was used to evaluate of the selection strength respectively and the *p* value of Chi square (χ^2^) smaller than 0.05 is thought as significant. The Bayes Empirical Bayes (BEB) inference [78] was implemented in site models M2a and M8 to estimate the posterior probabilities and positive selection pressures of the selected genes.

### Phylogenetic analyses

The phylogenetic analyses of 20 *Zingiber* species were performed based on chloroplast genomic data. The Maximum Likelihood (ML) method in Geneious R11 was used to construct the phylogenetic tree with default settings including 1000 bootstrap replications and the general time-reversible model with a gamma distribution of substitution rate among sites (GTR + G). In addition, Bayesian Inference (BI) was performed using MrBayes v3.2 [79], using the substitution model GTR and running parameters were as follows: the Markov Chain Monte Carlo algorithm was applied for 2 million generations with four Markov chains and sampled of trees every 100 generations, then the first 10% of trees were discarded as burn-in. The software Figtree v1.4 was used to edit and visualize the final BI tree and ML tree (Fig. 8). In addition, to clarify the phylogenetic position of *Zingiber* within the Zingiberaceae, we constructed a maximum likelihood tree based on chloroplast genome dataset of 55 Zingiberaceae species.

## Supplementary Information

**Additional file 1: Fig. S1.** Molecular phylogenetic tree based on 55 chloroplast genomes of Zingiberaceae. Species name in red color represent chloroplast genome obtained in this study. **Table S1.** List of RNA editing sites in fourteen Zingiber species by PREP program. **Table S2.** List of 14 species of Zingiber sequenced in this study.

## Data Availability

The complete chloroplast genomes generated during the current study were deposited in NCBI database (Accession number: OP869975, OP869976, OP869977, OP869978, OP869979, OP869980, OP869981, OP869982, OP869983, OP869984, OP869985, OP869986, OP869987, ON646165).

## Abbreviations

bp: Base pairs
ITS: Internal transcribed spacer
BI: Bayesian Inference
CNS: Conserved non coding sequence
dN: Non synonymous
DNA: Deoxyribonucleic acid
dS: Synonymous
IR: Inverted repeat
LSC: Large single copy region
SSC: Small single copy region
ML: Maximum Likelihood
RSCU: Relative synonymous codon usage
SSR: Simple sequence repeats
