# Correction: Complete chloroplast genomes provide insights into evolution and phylogeny of *Zingiber* (Zingiberaceae)

**DOI:** 10.1186/s12864-023-09393-3

**Published:** 2023-07-14

**Authors:** Dongzhu Jiang, Xiaodong Cai, Min Gong, Maoqin Xia, Haitao Xing, Shanshan Dong, Shuming Tian, Jialin Li, Junyao Lin, Yiqing Liu, Hong‑Lei Li

**Affiliations:** 1 College of Landscape Architecture and Life Science, Chongqing University of Arts and Sciences, Yongchuan, 402160 China; 2 College of Horticulture and Gardening, Yangtze University, Jingzhou, 433200 China; 3 College of Biology and Food Engineering, Chongqing Three Gorges University, Wanzhou, 404100 China; 4 Fairylake Botanical Garden, Shenzhen & Chinese Academy of Sciences, Shenzhen, 518004 China

**Correction: BMC Genomics 24, 30 (2023)**

**https://doi.org/10.1186/s12864-023-09115-9**

Following the publication of the original article [1], the authors reported that the 2^nd^ author was assigned affiliation 1 erroneously. Xiaodong Cai’s affiliation is presented correctly in this correction article.

Moreover, the authors identified an error in Fig. 3. The correct figure is given below.

**Fig. 3:**
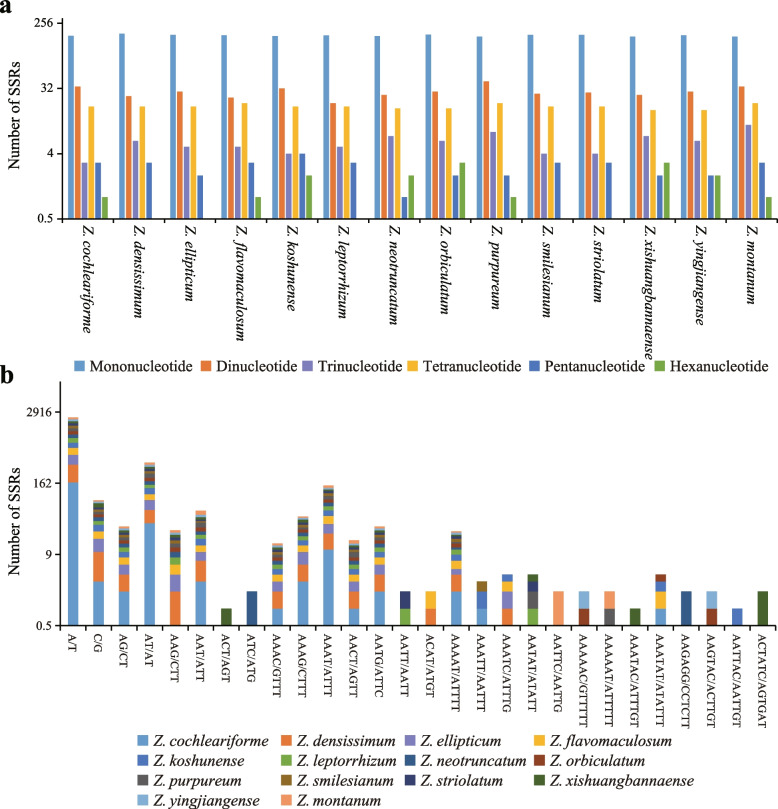
Comparison of the simple sequence repeats (SSRs) among fourteen *Zingiber* species. **a** The number of different SSR types. **b** The frequency of the identified SSRs in different repeat class types

The original article [1] has been updated.
