# Supplementary material for: Complete chloroplast genomes provide insights into evolution and phylogeny of Zingiber (Zingiberaceae)
Source: BMC Genomics. 2023 Jan 18;24:30. doi: 10.1186/s12864-023-09115-9 (PMC9848714; doi:10.1186/s12864-023-09115-9)
Supplement: Supplementary file 1 — Additional file 1: Fig. S1. Molecular phylogenetic tree based on 55 chloroplast genomes of Zingiberaceae. Species name in red color represent chloroplast genome obtained in this study. Table S1. List of RNA editing sites in fourteen Zingiber species by PREP program. Table S2. List of 14 species of Zingiber sequenced in this study. [file 12864_2023_9115_MOESM1_ESM.zip › (Supplementary material)Table S1, Fig S1 and Table S2/Fig. S1 Molecular phylogenetic tree based on 55 chloroplast genomes of Zingiberaceae.pdf]

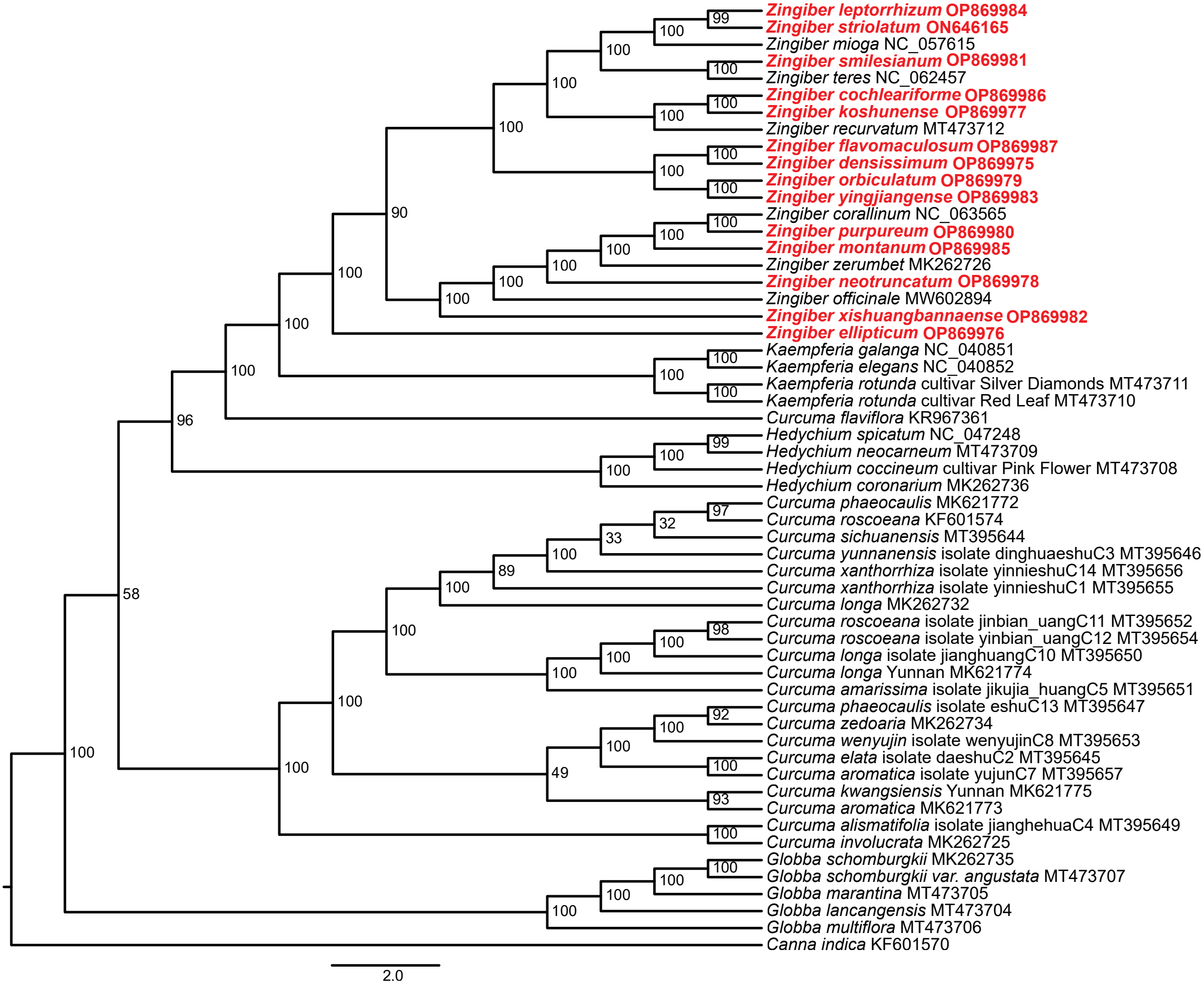

Fig. S1 Molecular phylogenetic tree based on 55 chloroplast genomes of Zingiberaceae. Species name in red color represent chloroplast genome obtained in this study.
